# Eco-Friendly Approach for the Recovery of Lactic Acid by Complex Extraction

**DOI:** 10.1021/acsomega.3c07988

**Published:** 2024-04-03

**Authors:** Aybikenur Erdas, Mustafa Esen Marti

**Affiliations:** † Department of Chemical Engineering, Konya Technical University, 42075 Konya, Turkey

## Abstract

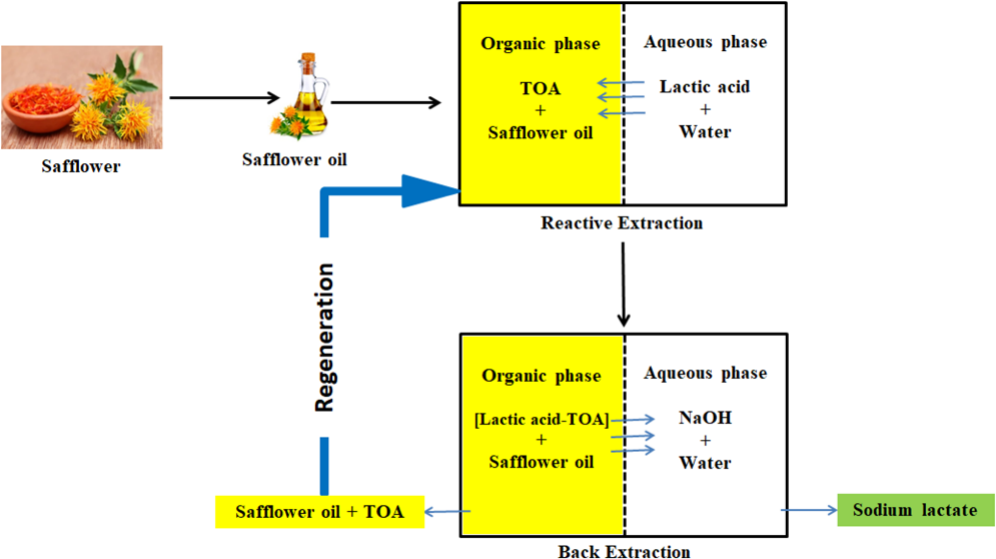

To meet the growing demand for high-purity lactic acid (LA) for biocompatible and biodegradable polymers, LA recovery by green techniques has been attracting the attention. This study focuses on the evaluation of vegetable oils as organic phase diluents in complex extraction of LA with an aliphatic tertiary amine extractant, trioctylamine (TOA). Eight vegetable oils were tested, and their performances were evaluated individually and compared with those obtained using 1-octanol. Extraction yields with these oils were similar; however, efficiencies with safflower oil (SFO) were slightly higher than those obtained with other oils tested. Efficiency with SFO + TOA varied inversely with temperature and pH; however, it increased with higher LA and TOA concentrations. Within the ranges of parameters investigated, the highest yield in SFO was 66% and was achieved at the highest TOA (1.0 M) and LA (1.5 M) concentrations. The efficiency obtained in 1-octanol under the identical conditions was 76%. Thus, the yields obtained with SFO + TOA and 1-octanol + TOA were comparable under most of the conditions tested, especially at the higher LA concentrations, which is preferred for commercial production. Following that, >99% of the LA was transferred from the organic phase to the (second) aqueous phase using NaOH (1.0 M) as a stripping agent. The organic phase was tested in subsequent extractions, and yields comparable to those obtained in the first uses were achieved. This study demonstrated that vegetable oils have the potential to be used as organic phase diluents during complex extraction of LA.

## 1 Introduction

Lactic acid (LA) has many industrial uses, such as in foods and beverages, cosmetics, pharmaceuticals, agriculture, polymers packaging, electronics, textiles, and personal care products.^1^ In recent years, its use as a monomer in the manufacture of biodegradable, biocompatible, and eco-friendly polyesters has grown. These biomaterials are already being used in sutures, implants, tablets, packages, fibers, resins, and foams.^2−4^ In the near future, they are expected to replace a significant number of conventional plastics in several industrial applications. The global market for LA is expected to exceed $6 billion by 2024, nearly half of the total organic acid market.^3^

Lactic acid (2-hydroxypropanoic acid) can be manufactured by chemical and biological methods from carbon-based resources.^5,6^ The former is not eco-friendly and uses relatively nonrenewable raw materials such as lactonitrile and hydrogen cyanide, which is also toxic.^7^ In addition, optically pure D-LA or L-LA isomers, which are preferred for industrial applications (e.g., polymers, foods, and pharmaceuticals), can be produced via microbial fermentation in biorefineries using low-cost feedstock or biomass.^2^ Biosynthesis of LA is carried out by bacteria, e.g., lactic acid bacteria (LAB, *Lactobacillus* sp.), *Bacillus* sp., *Escherichia coli*, *Corynebacterium glutamicum*, as well as filamentous fungi (e.g., *Rhizopus oryzae*), yeast, and microalgae.^8−10^ These organisms markedly reduce LA production costs by utilizing renewable low-value biomass such as lignocellulosic and agricultural wastes, food residues, dairy byproducts, and glycerol as feedstocks,^11,12^ thereby providing a great advantage for the remediation and valorization of low-cost byproducts and biomass wastes. Hence, >90% of LA used in industry is produced by fermentation.^4^ However, fermentation media are complex solutions that contain carbohydrates, proteins, salts, and other solutes in addition to the desired chemical(s) synthesized by the fermenting organism.^12−14^ Consequently, LA so produced requires an efficient, inexpensive, and also eco-friendly recovery process from a dilute solution. In industry, LA is precipitated as calcium lactate by using calcium hydroxide or calcium carbonate, and after filtration, the precipitate is acidified with sulfuric acid to convert Ca-lactate into soluble LA. Overall, the process is energy-intensive and expensive; it also produces large amounts of gypsum (calcium sulfate), resulting in expensive disposal problems. Furthermore, possible isomerization of LA during isolation can reduce the purity of the final product.^6,15^

According to the literature, separation and purification steps account for ∼50% of the cost of LA production^16−18^ and critically affect the price of the final product. Alternative recovery strategies include solvent extraction, ion exchange, distillation, adsorption, and electrodialysis.^13−19^ Conventional or bipolar membrane electrodialysis has been shown to be an effective method for purifying LA from fermentation broth; however, there remains a compatibility problem for the simultaneous operation of fermentation and electrodialysis processes.^5,18^ Furthermore, the cost of membranes and the reduction in permeate flow rate are significant drawbacks that hinder the cost-effectiveness of the process.^19^ Recovery of LA by adsorption or ion exchange using reusable resins has been tested and proposed by various researchers; but low yields and selectivity are the main disadvantages of the process that need to be improved.^6^ Complex(ation) extraction is an affinity based extraction technique and has shown to be one of the key separation processes for recovering various types of chemicals from dilute solutions.^20^ It is an attractive technique due to its low energy demand, process simplicity, low-cost, controllable selectivity, and high efficiency at optimum conditions.^21−24^ Moreover, the stability of the target chemical is not affected by heat since the process is generally carried out at room temperature.^25−27^ The method has been tested for several years for the extraction of value-added products such as amino acids, amines, alcohols, aldehydes, vitamins, antibiotics, phenols, metals, enantiomers, and carboxylic acids.^16,20,28^ The desired product, e.g., LA, is extracted from an aqueous production medium (i.e., fermentation) to an organic phase in step 1 (complex extraction) and then transferred to another (second) aqueous phase in step 2 (stripping or back extraction). The choice of extractant-diluent pairing for the organic phase is critical to maximize efficiency and minimize costs and toxicity risks.

Separation of carboxylic acids (e.g., LA) from aqueous media by complex extraction has been well researched.^20,24^ Although a viable alternative for the purpose, the technique suffers from the toxicity of the chemicals used in the system.^28^ Therefore, efforts have focused on eliminating or reducing the toxicity of the organic phases employed in the process, which has gained importance, especially due to organic chemical pollutants released from industry to the environment. According to earlier reports, replacement of extractant in the extraction system is more arduous compared to that of the diluent. As a result, vegetable oils have been tested as organic phase diluents for the process.^28−36^ These have the advantage of being eco-friendly, renewable, nontoxic, inexpensive, nonvolatile, and immiscible with water. Their availability for complex extraction would also be useful for the extractive fermentation, in which simultaneous production and separation of the product is possible.^37^ Previously, in order to recover LA from a solution having a concentration of 0.2 M, Aliquat 336 and trioctylamine (TOA) were used as extractants, and tributyl phosphate and sunflower oil (35 wt %) served as diluents in the organic phase and 70% efficiency was achieved. Several studies have shown that acid concentration is a critical parameter affecting extraction efficiency, and higher LA concentrations (>0.5 M) are recommended to have an effective and applicable process.^5,13,14^ In addition, mixed extractant systems are often arduous to control in industrial applications, particularly in fermentation media. A butanol-modified organic phase containing vegetable oils and extractant mixtures including Aliquat 336, tri-n-dodecylamine, and TOA was used to recover LA.^31^ However, earlier studies have shown that selecting an appropriate “vegetable oil + extractant” blend will maximize recoveries and eliminate the need for multi extractant systems and a modifier, which also increases the toxicity and cost of the organic phases.^32^ Moreover, the use of butanol, which is partially miscible with water (73 g/L at 25 °C), may adversely affect the separation of phases after reaching equilibrium and compromise the acid transfer. Lastly, to evaluate these nontoxic alternatives for LA recovery by complex extraction, the efficiencies obtained with vegetable oils should be compared with those provided by solvents currently in use, e.g., 1-octanol under the same conditions.

The focus of this study is the evaluation of eight vegetable oils as organic phase diluents during the complex extraction of LA, mainly to determine whether these natural, nontoxic, and inexpensive alternatives can be used in the extraction process, compare their performances with an active diluent, such as 1-octanol, which has shown to be one of the most efficient diluent in the literature, and observe the effects of the parameters, e.g., pH, temperature, and concentration (LA and TOA), when these eco-friendly diluents are used in the system.^20−24^ Tertiary amines have shown to be effective alternatives for the purpose;^15,25−28^ therefore, TOA was used as the extractant. In addition, the LA extracted to the organic phase was recovered to another (second) aqueous phase using several stripping agents, and the suitability of the regenerated organic phase for reuse was tested. The results were used to observe the effect of using vegetable oils in the process in these steps. The effect of using vegetable oil in the complex extraction on stripping and reuse was investigated.

## 2 Theory

Lactic acid is a weak acid (*K*_A_ = 8.3 × 10^–4^, p*K*_A_ = 3.86) and dissociates in an aqueous medium according to the solution pH (eq 1). During its physical extraction (without use of an extractant), undissociated lactic acid (HL) can partition between the phases (eq 2) and may form dimers in the organic medium depending on its concentration and organic solvent used in the system (eq 3).^26^
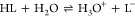
1
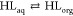
2
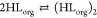
3

Tertiary amines, e.g., TOA, are the most extensively examined extractants, having a single electron pair on the nitrogen atom that is coordinated with the acid molecule through dipole–dipole interactions. The strength of the bond is dependent upon the stability of the partially charged proton donors and acceptors.^22,25,38^ If the extractant has a greater basicity than the anion of the solute, complex extraction of protonated carboxylic acids with aliphatic tertiary amines involves the ion pair formation between these molecules and transfer of the target molecule to the organic phase (eq 4)^39−41^ and the complexation constant can be calculated using eq 5. Thus, concentration of the undissociated or protonated acid portion in the aqueous medium plays a key role in the extraction with aliphatic amines.^28,42^ Extraction performance of the process was evaluated by the extraction efficiency (*E* (%)) and distribution coefficient (*K*_D_) as expressed in eqs 6 and 7, respectively.
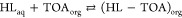
4
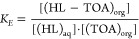
5
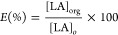
6
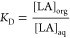
7

The stability or solubility of the acid–amine complex strongly depends on the organic phase diluent and tends to improve as the polarity of the diluent increases. The stoichiometry of the acid–amine complex can be estimated by using the loading ratio (*z*), which is the ratio of the concentration of the acid complexed with the extractant to the total amine concentration in the organic phase (eq 8). If the stoichiometry between acid and amine is 1:1, an acid and amine molecule bind to form a 1:1 acid–amine complex (eq 4). Previous reports showed that binding more acids to an amine molecule (z > 1) may result in the formation of (n:1, *n* > 1) complexes in the organic phase (eq 9).^25^
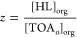
8
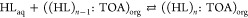
9

The concentration of protonated acid in the aqueous phase depends on the solution pH, p*K*_A_, and initial molarity of the corresponding acid (eq 10).^27,39^ Since the addition of acid or base molecules to the aqueous solution changes the pH of the medium, the extraction of the target acid with amine extractant is also expected to be affected. Another process variable, temperature may also affect the distribution of a carboxylic acid between an organic and aqueous media due to the exothermic nature of the acid–amine complexation.^43,44^
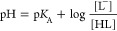
10

The acid transferred to the organic phase must be recovered from this medium. Contacting an aqueous solution having a pH higher than the p*K*_A_ of the target acid with the organic phase containing the acid–amine complex triggers the removal of the acid, resulting in its transfer from the organic phase to the aqueous medium.^28,45^ Several reports have recommended using aqueous solutions of sodium salts and alkaline agents during the stripping of carboxylic acids from organic media, and the process with these substances follows the displacement reaction (addition of salt, eq 11) and the pH-shift (eq 12) mechanisms, respectively.^46−48^ Stripping efficiency (S(%)) can be calculated from eq 13.
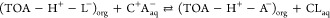
11
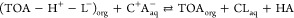
12
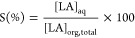
13

## 3 Experimental Section

### 3.1 Materials

Vegetable oils (sunflower, safflower, sesame, soybean, corn, almond, hazelnut, and canola) purchased from local markets were individually tested as organic phase diluents and were used without pretreatment. The extractant, trioctylamine (TOA), and active diluent, 1-octanol, were supplied by Acros Co. Lactic acid (LA; 90% purity) was supplied by Merck Co. This was heat-treated before use to hydrolyze LA-esters and to dissociate dimers that form at [LA] > 20 wt %.^37,39^ Ultrahigh pure (UHP) water obtained from a Millipore Direct-Q 3 V system was used throughout this study. NaOH (≥99% purity) or HCl (37% purity) was used to adjust aqueous pH. Moreover, NaNO_3_, NaCl, Na_2_SO_4_, NaOH, and Na_2_CO_3_ were evaluated as stripping agents and all were supplied by Merck Co.

### 3.2 Extraction Method

Aqueous LA solutions were prepared in UHP water; TOA was dissolved in vegetable oils or 1-octanol to obtain the organic phases. Kinetic experiments were carried out to determine the equilibration time at 298 K and 150 rpm. The volume ratio of the phases (*V*_org_/*V*_aq_) was fixed at 1:1 throughout the study. The initial LA concentration varied between 0.1 and 1.5 M to mimic the LA fermentation medium. In experiments where the initial amount of acid had to be kept constant, the LA concentration was adjusted to 0.5 or 0.6 M around the midpoint of the range. The TOA concentration ranged from 0.2 to 1.0 M (∼8.7–43.5% (v/v)) to keep the volume of the vegetable oil higher than that of the amine in the organic phase. However, the effect of amine concentration in the organic phase was also investigated at extreme levels of TOA, 1.5 and 2.0 M, where organic phase mostly consists of the extractant. Initially, physical extraction trials were done, followed by complex extraction experiments. The effects of pH and temperature were tested over the ranges of pH 1–6 and 298–318 K, respectively. Extractions were performed by mixing organic and aqueous phases in 50 mL Erlenmeyer flasks at 25 °C (298 K) and shaking at 150 rpm for 1 h in a constant temperature shaker bath. The mixtures were then centrifuged at 4000*g* for 15 min and settled for 60 min to produce clear phase separations. Aqueous phases were recovered to determine the residual LA concentration.

### 3.3 Stripping and Reuse

The stripping agents were dissolved in UHP water to prepare their aqueous solutions for use in the back extraction of LA from organic phases. Similarly, the volume ratio of the phases was set at 1:1. Experiments were performed by mixing the organic media obtained from complex extraction trials (containing the LA-TOA complex) with aqueous solutions of stripping agents in 50 mL Erlenmeyer flasks at 298 K and 150 rpm for 1 h. The amount of LA extracted into the aqueous medium upon equilibration was calculated from analysis of the aqueous phases. Regenerated (LA-free) organic phases were reused in the subsequent complex extraction trials, and yields were compared to observe the effect of reuse.

### 3.4 Assay Method

The concentration of aqueous LA before and after each extraction step was determined by HPLC (Agilent LC 1220) equipped with an ACE 5 AQ C_18_ column (250 × 4.6 mm, ACE). The mobile phase was 50 mM potassium dihydrogen phosphate (KH_2_PO_4_) + 1% acetonitrile solution, pH 2.8. HPLC analyses were performed at 30 °C and 1.25 mL/min flow rate; LA was detected at 210 nm using a UV detector. All experiments and analyses were performed in duplicate, and the results were averaged to calculate the extraction parameters. The experimental uncertainty (*u*) or variation of the observation from the mean was calculated to vary within the range of 0.001 < *u* < 0.01.^38^ The concentration of LA in the organic phase was calculated by a mass balance in the aqueous phase.

## 4 Results and Discussion

Before the effects of system variables on extraction were assessed, kinetic experiments were performed to ascertain the equilibration time for the complex extraction of LA with TOA dissolved in vegetable oils and 1-octanol. The concentrations of LA and TOA were held constant at 0.6 M. The organic phases containing 1-octanol and vegetable oils reached equilibrium after 45 min at 298 K and 150 rpm. Similar profiles and equilibration times were achieved for the vegetable oils tested.

### 4.1 Effect of Diluent Type

In this study, eight vegetable oils were evaluated for the extraction of LA from an aqueous medium. Preliminary trials using vegetable oils revealed that recovering LA by physical extraction (without TOA) was either impossible or produced negligibly low yields (data not shown). This is most likely due to the hydrophobic and nonpolar nature of the vegetable oils. They are lipophilic substances with variable and complex compositions. In the next step, these vegetable oils were tested as organic phase diluents for separating LA from aqueous phases by complex extraction, and their relative efficiencies were compared. In these experiments, the concentration of amine extractant in the organic phase was kept constant at 0.6 M and the LA molarity ranged from 0.3 to 1.0 M. Thus, the initial pH of the aqueous phase varied between 1.8 and 2.2. The phases were equilibrated by shaking at 298 K and 150 rpm for 1 h.

Although vegetable oils alone in the organic phase could not extract carboxylic acids, their mixtures with TOA recovered LA from the aqueous phase.^28,29^ In the first trials, the LA concentration was 0.3 M and the yields achieved with vegetable oils were almost the same or negligibly different from each other (Table 1). To confirm the result, additional trials were carried out at LA concentrations of 0.5 and 1.0 M. The trend continued and almost similar yields were obtained in different oils.

**Table 1:** Efficiencies Obtained in Different Vegetable Oils for the Complex Extraction of LA with TOA ([TOA]_o_ = 0.6 M, [LA]_o_ = 0.3–1.0 M, pH 1.8–2.2, T = 298 K, 150 rpm, 1 h)

	*E* (%)		
[LA]_o_	0.3 M	0.5 M	1.0 M
sesame oil	27.5	33.1	43.6
safflower oil	27.8	33.3	43.8
sunflower oil	27.4	32.9	43.5
soybean oil	23.5	30.7	42.6
corn oil	24.2	31.0	42.5
almond oil	26.7	30.6	42.3
hazelnut oil	24.3	32.0	41.5
canola oil	24.2	31.2	41.8

The principle (95–98%) building blocks of these oils are triglycerides whose fatty acid makeup varies depending on the origin, climate, quality, cultivation, and production methods of the plant source. Unsaturated C-16 and C-18 fatty acids are the most common in vegetable oils. Fatty acids may positively affect the stability of the acid–amine complex in the vegetable oil.^33^ Glycerolipids, phospholipids, vitamins, proteins, phenolics, and water are some of other components of oils. Water and some phenolic compounds are the only polar compounds in vegetable oils; however, their percentages are <0.1%. Moreover, the dielectric constants of these oils are also similar.^49,50^Table 1 indicates that these vegetable oils can be used to recover LA from aqueous solutions, and the data also show that the type of oil slightly affects the complex extraction efficiency. The yields with these vegetable oils were very similar, most likely due to the similar hydrophobic nature of these oils or their minor compositional variations. However, Table 1 shows that the yields with safflower oil (SFO) were slightly or insignificantly higher than the efficiencies obtained with the other oils tested. In addition, sunflower oil and sesame oil produced nearly identical yields. Sunflower oil is the main cooking oil in many countries. Compared to sunflower oil and safflower oil, sesame oil is relatively expensive. Therefore, SFO was selected as the representative organic phase diluent or vegetable oil to further investigate the effects of process parameters and evaluate the applicability of these eco-friendly alternatives for LA recovery.

### 4.2 Effect of Aqueous pH

**Figure 1:**
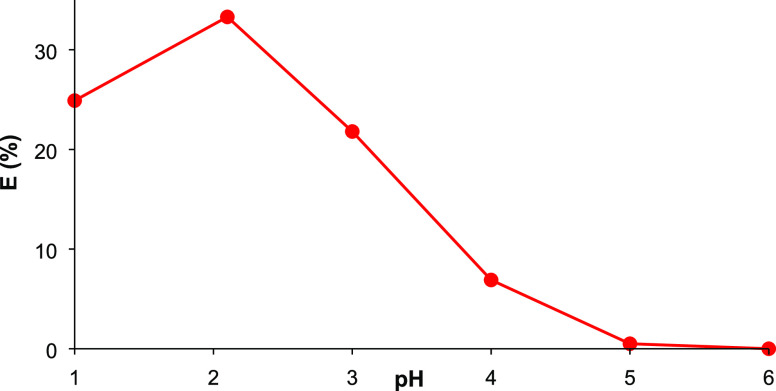
Effect of initial pH on complex extraction of LA using TOA in SFO ([TOA]_o_ = 0.6 M [LA]_o_ = 0.5 M, pH 2.1, *T* = 298 K, 150 rpm, 1 h).

Figure 1 shows the effect of solution pH on the extraction of LA by TOA dissolved in SFO. The concentrations of LA and TOA were 0.5 and 0.6 M, respectively. The initial pH of the aqueous solution ranged from pH 1 to 6 and was adjusted using NaOH and HCl. The highest efficiency occurred at the original or unadjusted pH (pH 2.1) of the aqueous LA solution containing predominantly (>98%) undissociated LA. Although the concentration of protonated LA was higher at pH 1, the yields were lower than those obtained at pH 2.1. Tertiary amines, i.e., TOA, preferably reacts with the strongest acid in the medium, in this case, HCl.^42,44^ Since the concentration of HCl in the solution was relatively low, only *a* < 10% decrease was observed at pH 1.

Harington and Hossain used TOA, Aliquat 336, and their mixtures for LA recovery in the range of pH 4–6. They observed that extraction yields reduced with the increase in aqueous pH for all organic systems tested.^29^ Consistently, increasing the initial pH of the aqueous solution resulted in decreased efficiencies with TOA in SFO (Figure 1). There was almost no LA transfer at pH 5 and 6, most likely due to the extremely low concentration of protonated acid in the aqueous phase.^35,39^ A similar profile was observed when 1-octanol was used as the diluent in the organic phase (data not shown). The trend is in agreement with the results previously reported in the literature.^27,28^ Various reports have mentioned that the trend is due to the decrease in undissociated acid concentration in the aqueous medium.^35^ As previously stated, tertiary amines cannot interact and extract dissociated acids. Hence, initial pH of the aqueous medium (i.e., the ratio or concentration of undissociated LA in the aqueous phase) is critical to achieve an efficient recovery with TOA (*p* < 0.05, *f* > 5).^21−24^

The final pH of the LA fermentation medium has been shown to be significantly higher than its p*K*_A_ value.^8−12^ Hence, the pH of the aqueous-based bioproduction medium needs to be lowered before the extraction step to achieve a high separation efficiency. Moreover, it was noted that the equilibrium pH of the aqueous medium may deviate due to the TOA impurities such as primary and secondary amines. Therefore, the pH of the aqueous phase was measured after extraction and observed to be higher than the initial pH of the solution. It was nearly the same as the theoretical pH (according to the residual acid concentration in the aqueous phase) regardless of diluent type and TOA concentration (data not shown), indicating no interference from vegetable oils and commercial tertiary amine.^25,45^

### 4.3 Effect of Temperature

The importance of the temperature as a critical process parameter stems from its capacity to influence the solubility and interaction or reactivity of chemicals in a liquid medium. Both solvation and acid–amine complex formation via proton transfer exhibit exothermic behavior.^43,51^ Hence, elevating the temperature results in a decrease in the extraction of organic acids in most of the systems that were examined.^28,38,52,53^ In this study, the effect of temperature on LA extraction by using TOA in SFO was investigated in the range of 298–338 K. The concentration of TOA ranged from 0.2 to 1.0 M, while that of LA was fixed at 0.5 M.

**Figure 2:**
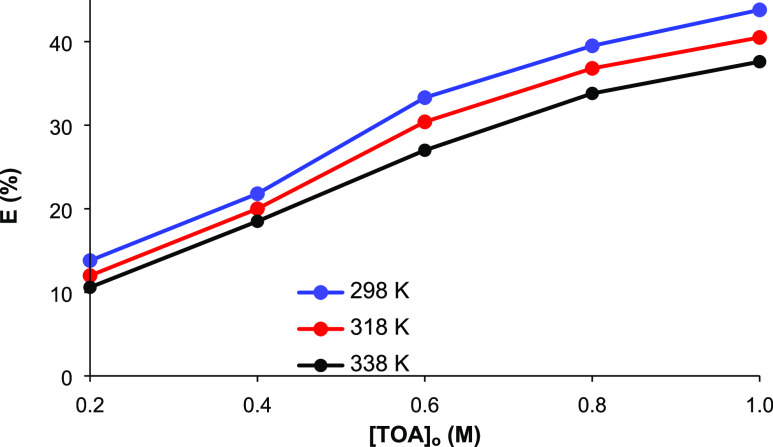
Effects of temperature and TOA concentration on the complex extraction of LA using TOA in SFO ([LA]_o_ = 0.5 M, pH 2.1, [TOA]_o_ = 0.2–1.0 M, 150 rpm, 1 h).

Figure 2 shows that separation efficiencies at 298 K were slightly higher than those at 318 and 338 K at all TOA concentrations tested, showing that extraction performance varies inversely with temperature. Consistent with previous studies, the trend observed for the complex extraction of LA by TOA in SFO confirms the exothermic nature of the separation process.^42,52^ Increasing thermal energy of the extraction system may damage acid–amine interactions, resulting in lower recoveries.^28,43^ The decreasing trend may be influenced by the dielectric constant (i.e., polarity) of the organic medium, which decreases with increasing temperature.^49,50^ Previously, lowering the temperature from 298 to 288 K also enhanced the recovery of gallic acid by complex extraction, regardless of the type of extractant used.^38^ The decrease in extraction performance with increasing temperature may provide an advantage for stripping the target molecule from the organic phase.^20^ However, the superiority was not notable, and the yields obtained at lower temperatures were negligibly higher than those at higher temperatures for LA extraction. Consistent with this, many earlier reports have also shown the insignificant influence of temperature on extraction efficiency, especially for hydrophilic acids.^25,27^ Hence, the separation of LA from aqueous-based media by complex extraction can be performed at low temperatures or room conditions without adjusting the temperature to keep the separation cost as low as possible.

### 4.4 Effect of Concentration

While the impacts of process parameters were examined in previous sections, the LA and/or TOA concentrations were chosen around the midpoint of the concentration ranges. However, both acid and extractant concentrations have significant effects on the process yield. In prior reports, the LA concentration was kept very low or studied in a limited range. In the trials to investigate the effects of concentrations of TOA and LA, the LA molarity ranged from 0.1 to 1.5 M to be able to test the parameter over a wide range using titers from literature reports and to mimic an effective fermentation medium.^5−15^ The pH values of the aqueous phases were in the ranges of pH 1.8–2.2. TOA concentration was chosen between 0.2 and 1.0 M (∼8.7–43.5% (v/v)) to keep the volume of vegetable oil in the organic phase higher than that of the amine extractant. As an exception to this section, the influence of extractant concentration was also investigated at the extreme TOA levels of 1.5 and 2.0 M, where the organic phase consisted mostly of the tertiary amine agent. Mixing was done at 298 K and 150 rpm for 1 h.

**Figure 3:**
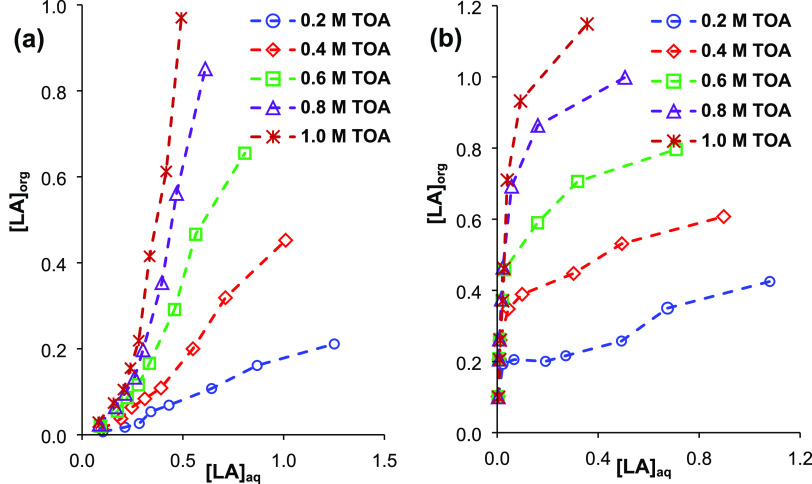
Equilibrium isotherms for complex extraction of LA with TOA dissolved in (a) SFO and (b) 1-octanol. ([LA]_o_ = 0.1–1.5 M, [TOA]_o_ = 0.2–1.0 M, and *T* = 298 K, pH 1.8–2.2, 1 h).

Although most of the studies testing the suitability of vegetable oils for the extraction process have compared the yields obtained with different oils, their performance should also be compared with the yields obtained with the organic solvents recommended in the literature. Equilibrium isotherms for the complex extraction of LA using TOA in SFO and 1-octanol are shown in Figure 3. The variation of the LA concentrations in the organic phase with that in the aqueous phase differs for the two diluents tested, especially at low aqueous molarities, indicating different influences of concentrations in SFO and 1-octanol. A very sharp increase at low acid concentrations in the aqueous phase is indicative of high distribution coefficients and extraction efficiencies in 1-octanol; the decreasing slopes at higher LA molarities may signify the saturation of the organic phase. In SFO, a linear-like relationship between the concentrations and relatively low slopes were observed at low TOA molarities (0.2–0.6 M). The slopes became steeper at higher TOA concentrations, indicating higher extraction yields.^39−43^

**Figure 4:**
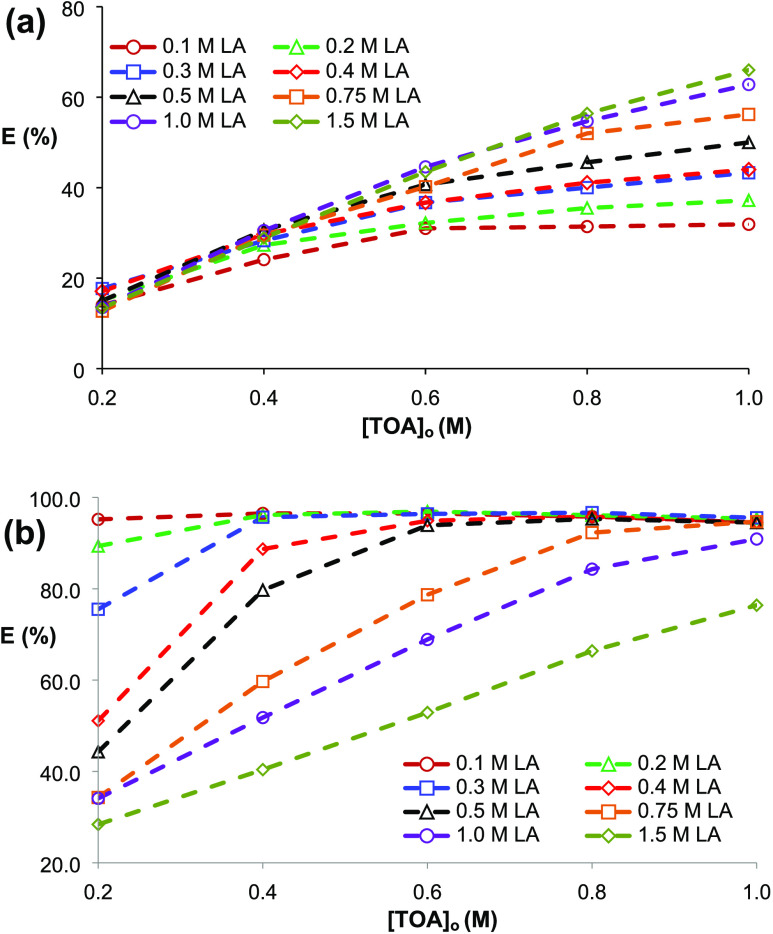
Effects of initial acid and extractant concentrations on the complex extraction of LA using TOA from aqueous solutions (a) SFO and (b) 1-octanol.

Figure 4 shows that acid and extractant concentrations significantly affect the extraction efficiency (*p* ≪ 0.05, *f* > 5). For both diluents, TOA concentration has a positive effect on the extraction yield in the range of 0.2–1.0 M TOA. For 1-octanol, the effect of extractant concentration could not be clearly observed at LA molarities ≤0.3 M because of the extremely high yields over this TOA range (0.2–1.0 M). At higher TOA levels, where amine extractant has a higher volume percentage than the diluent in the organic phase, extraction yields obtained at 1.5 and 2.0 M TOA were similar, and the trend was detected for both diluents tested. This may be due to the saturation of the organic phase or TOA molecules with LA molecules. In SFO, the effect of LA concentration was not obvious at TOA molarities of ≤0.4 M; however, at higher extractant levels (≥0.6 M), yields clearly increased with the increase in LA concentration (Figure 4a). Previously, it was also shown that the distribution coefficient increased with increasing initial LA concentration in the leached bed reactor leachate.^54^ A similar trend was observed for the complex extraction of propionic acid and acrylic acid from aqueous solutions using natural diluents as organic phase diluents.^32,33^ However, when 1-octanol was used in the organic phase, the efficiency was reduced as the amount of acid in the aqueous phase increased (Figure 4b). The trends were consistent with the previous complex extraction reports carried out using vegetable oils and alcohols.^25−36^

In the ranges of the parameters studied (0.2–1.0 M TOA and 0.1–1.5 M LA), the maximum efficiency in SFO was 66% (*K*_D_ = 1.94) and obtained at the highest TOA and LA molarities; under the identical conditions, the percent recovery achieved in 1-octanol was ∼76% (*K*_D_ = 3.24) (Figure 4). These yields are consistent with efficiencies reported in the literature for the complex extraction of LA.^22,26,39,51−53^ The yield in SFO enhanced to 80.6% (*K*_D_ = 4.15) by increasing the TOA concentration (to 2.0 M); but as previously mentioned, this is unfavorable since the organic phase primarily contains amine extractant (Figure S1). On the other hand, the maximum efficiency of TOA + 1-octanol was obtained as ∼96–97% at ≤0.3 M LA levels, depending on the TOA concentration (Figure 4b).

In general, the trends observed for the complex extraction of LA with SFO + TOA or 1-octanol + TOA are consistent with the literature reports using vegetable oils or alcohols as organic phase diluents. The efficiencies obtained with TOA in SFO were either comparable or higher than those achieved with organic phases using a (toxic) modifier agent or mixed extractant systems.^22−36^ The physical and chemical properties of the components in the system affect the interactions between the extracted acid, amine, acid–amine complex, and diluent. Hydrophobicity, polarity, chemical structure, and composition of SFO and 1-octanol differ from each other, likely accounting for the different trends observed with these diluents. Reportedly, high extractant concentrations increase emulsion formation at the interface that retains a significant amount of the target product and is difficult to disrupt by centrifugation or emulsifiers; however, the formation of a third phase was not observed in this study. The higher viscosity of vegetable oils vs conventional organic solvents can be considered as a critical issue; however, no significant difference was observed in phase separation with 1-octanol and vegetable oils (e.g., SFO) for LA extraction at the concentrations tested.

**Figure 5:**
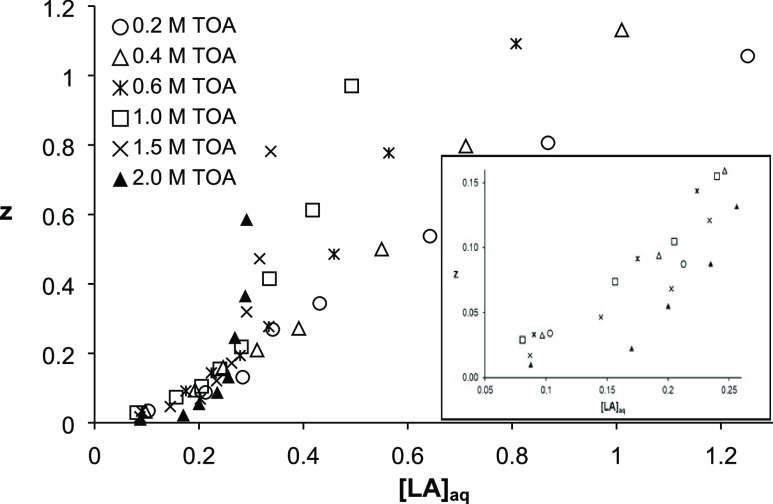
Variation of the loading ratio with initial acid and extractant concentrations for complex extraction of LA by TOA in SFO ([LA]_o_ = 0.1–1.5 M, pH 1.8–2.2, [TOA]_o_ = 0.2–2.0 M, *T* = 298 K, 1 h).

Loading ratios specify the stoichiometry of the acid–amine complex between LA and TOA in the organic phase. As SFO cannot recover LA without TOA, all LA molecules in the organic phase are complexed with TOA through ion pair formation.^28,35,55^ The *z* value increased with the increase in equilibrium concentration of LA in the aqueous phase. When LA molarity was ≤0.4, the *z* values were <0.4, and the TOA concentration had no effect on the loading ratio, consistent with the literature.^27,44^ Although higher *z* values were obtained at higher LA concentrations, they were usually less than or close to unity (Figure 5). Hence, the stoichiometry of the LA-TOA complex formed in SFO was estimated to be 1:1.

### 4.5 Stripping

The data indicated that TOA + SFO produced yields comparable to those of TOA + 1-octanol; however, LA that complexed with TOA must be separated from the organic phase. A variety of techniques and approaches can be used to achieve this goal. The swing of process conditions, e.g., pH, temperature, and composition, has been tested in the literature. The back extraction of carboxylic acids by temperature swing has been extensively studied.^56,57^ However, due to the lack of temperature sensitivity in the distribution of LA, it alone is insufficient for the successful recovery of LA from the organic phase. It was observed that the back extraction of succinic acid extracted using a tertiary amine was more sensitive to pH than to temperature.^58^ Moreover, pyruvic acid extracted with TOA was successfully recovered from the organic phase via pH swing, and the highest yield was obtained when the molar ratio of stripping agent to acid was unity.^59^

Therefore, stripping or back extraction of LA using appropriate stripping reagents is an attractive option. This also regenerates the organic phase, which can be used in subsequent extractions. Most studies that used vegetable oils as organic phase diluents did not investigate the acid stripping step or the impact of the type of stripping agent. A few reports have used only one type of agent to recover acid from the organic phase. In this study, NaOH, NaCl, Na_2_CO_3_, NaNO_3_, and Na_2_SO_4_ were tested as the stripping agent for the recovery of LA from the organic phases containing TOA-LA in SFO or 1-octanol.^28−30^ Sodium sulfate was employed at 0.9 M due to its limited solubility in aqueous media; all other salts and bases were used at the concentration of 1.0 M. Stripping trials were carried out using the organic media obtained from complex extraction experiments. Separate runs were performed for SFO and 1-octanol to examine the effect of the organic phase diluent and appropriateness of vegetable oil for the process. The concentrations of LA and TOA in these (complex) extraction trials were 1.5 and 1.0 M, respectively; and the recovery yields under these conditions were ∼66 and ∼76% with SFO and 1-octanol, respectively. Therefore, the initial LA concentration was about 1.0–1.1 M in these organic phases.

Table 2 shows that all of the tested stripping agents could recover LA from the organic phase. The most effective agent was NaOH; however, relatively high yields were obtained with NaNO_3_ and NaCl. In complex extraction, tertiary amine and undissociated acid form an acid–amine complex, and the amount of protonated acid in the medium is so important, which is also dependent on medium pH. In stripping or back extraction, an aqueous solution with a higher pH (>p*K*_A_) is used, and this destroys the bonding between acid and amine in the complex structure. As a result, the target acid is released from the organic phase to the aqueous solution in its dissociated form. The transfer takes place through a pH-shift mechanism when an alkali agent such as NaOH or Na_2_CO_3_ is employed, and this has been reported to be more effective than the displacement reaction that occurs when a salt, e.g., NaCl, is used in the stripping process.^60^Table 2 shows that the efficiency decreased in the order NaOH > NaNO_3_ > NaCl > Na_2_SO_4_ > Na_2_CO_3_, regardless of the diluent type, and the data suggest that the mechanism and the anion type of the stripping agent play the main role in the process. Moreover, this order parallels the decreasing order of the dielectric constants of these stripping agents. This trend is consistent with previous results reported for the back extraction of carboxylic acids from organic phases containing tertiary amines as extractants.^45^ More than 99% of the LA in the organic phase was recovered using NaOH (1.0 M) and recoveries were almost identical for both SFO and 1-octanol, indicating that the use of SFO does not cause a problem for the separation of LA from the organic phase.

**Table 2:** Effects of Stripping Agent Type on the Back Extraction of LA from the Organic Phase to the Second Aqueous Phase (T = 298 K, 150 rpm, 1 h)

		stripping (back extraction) efficiency (%)	
stripping agent	concentration (M)	SFO	1-octanol
Na_2_SO_4_	0.9	43.2	43.5
NaCl	1.0	66.8	67.8
NaNO_3_	1.0	80.3	82.4
NaOH	1.0	99.4	99.9
Na_2_CO_3_	1.0	26.5	27.4

Following this result, the free fatty acid content of the system was examined due to the possibility of soap formation by adding NaOH to the system containing vegetable oil. It was found to be <0.1% before extraction and ∼0% after the process. Lactic acid is the strongest acid in this system and sodium ions react with the strongest acid in the media; therefore, soap formation did not occur and sodium salt of LA formed during the stripping step. Following complex extraction and stripping, crystallization or evaporation can be used to recover high-purity LA.

### 4.6 Organic Phase Reuse

Reusing the organic phase can significantly decrease the cost of the separation process. Nevertheless, the number of studies on this step of the process has been quite limited in the literature. In this section, LA-free organic phases that were produced during the stripping step were tested in the subsequent complex extraction processes. Table 3 summarizes the efficiencies obtained in consecutive complex extraction and stripping trials. The data showed that the organic phases can be reused in subsequent complex extractions. The reuse of the organic phase resulted in a ≤ 10% reduction in efficiency in each subsequent application. However, the efficiencies were nevertheless comparable to those achieved in the first uses. Stripping efficiencies changed negligibly in the subsequent stripping steps. The comparable recoveries obtained with SFO and 1-octanol demonstrated the potential use of SFO for the recovery of LA using tertiary amines via a complex extraction.

**Table 3:** Reuse of Organic Phases in the Subsequent Extraction Trials

	SFO		1-octanol	
cycle	complex extraction	stripping	complex extraction	stripping
	*E* (%)	*S* (%)	*E* (%)	*S* (%)
1st	66.0	99.4	76.4	99.9
2nd	61.6	99.2	69.8	99.8
3rd	56.7	99.1	65.1	98.6

## 5 Conclusions

In this study, an eco-friendly and cost-effective process has been proposed for the complex extraction of LA from aqueous solutions. Eight vegetable oils were tested, and their extraction efficiencies along with TOA for LA recovery were very similar; however, yields with safflower oil (SFO) were slightly higher than those obtained with other oils tested. The initial pH of the aqueous phase had a remarkable effect on extraction efficiency, and maximum yields were obtained at the unadjusted pH (pH 2.1 at 0.5 M LA) of the aqueous LA solution. The extraction efficiency in SFO reduced at higher temperatures, indicating the exothermic nature. The amine extractant concentration had a positive effect on the extraction yield for both diluents in the range of 0.2–1.0 M TOA. The trends observed with LA concentration varied depending on the type of diluent used in the organic phase. Extraction efficiencies in SFO increased at higher LA molarities; however, the effect was in the opposite direction with 1-octanol. In the concentration ranges examined, the highest yield in SFO was 66% and was achieved at the highest TOA and LA concentrations; under the identical conditions, the efficiency obtained in 1-octanol was 76%. The recoveries achieved with SFO + TOA and 1-octanol + TOA were comparable under most of the conditions tested, especially at the higher LA concentrations, which is preferred for commercial production. Following that, LA was back-extracted to the (second) aqueous phase. Various alternatives were tested, but NaOH (1.0 M) produced the highest yield, recovering >99% of the acid from the organic phase. The trend was observed for both diluents evaluated. The organic phase was tested in three subsequent extraction trials, and yields comparable to those obtained in the first uses were achieved. The data reinforce the viability of using SFO or another appropriate vegetable oil as an organic phase diluent for the recovery of LA from aqueous-based solutions. In the following step, testing the process with a laboratory-scale mixer–settler, membrane contactor, or extraction column would be beneficial to assess the practicability of scaling up and continuous operation. The use of fermentation media is important to test the feasibility of the recovery process. In addition, the evaluation of vegetable oils in the extractive fermentation of LA will also be useful to carry out simultaneous production and separation of the product. Phase separation in these systems should also be examined in the subsequent studies.

## Supporting Information Available

Effect of initial acid concentration on the complex extraction of LA at extreme TOA molarities (1.5 and 2.0 M) in SFO and 1-octanol (PDF)

## Notations

A^–^: anion of the stripping agent
C^+^: cation of the stripping agent
E (%): complex (forward) extraction efficiency
H^+^: hydrogen ion
HL: undissociated lactic acid
HL-TOA: lactic acid–trioctylamine complex
K_A_: acid dissociation constant, (M)
K_D_: distribution coefficient
K_E_: acid–amine equilibrium complexation constant (M^–1^)
L^–^: lactate ion (dissociated lactic acid)
LA: lactic acid, all forms in equations
S (%): stripping (back extraction) efficiency
SFO: safflower oil
TOA: trioctylamine
V: volume of the phase, mL
z: loading ratio, [LA] (M)/[TOA] (M)
[ ]: concentration (M)

## Subscripts

aq: aqueous phase
o: at initial (for concentration)
org: organic phase
