# Supplementary material for: Eco-Friendly Approach for the Recovery of Lactic Acid by Complex Extraction
Source: ACS Omega. 2024 Apr 3;9(15):16959–68. doi: 10.1021/acsomega.3c07988 (PMC11025082; doi:10.1021/acsomega.3c07988)
Supplement: Supplementary file 1 — ao3c07988_si_001.pdf [file ao3c07988_si_001.pdf]

An eco-friendly approach for the recovery of lactic acid by complex extraction

Aybikenur Erdas [a] *and Mustafa Esen Marti* [a,*]

[a] Department of Chemical Engineering, Konya Technical University, 42075 Konya, TURKEY
[*]Corresponding author: Prof. Dr. Mustafa Esen Marti, e-mail: memarti@ktun.edu.tr

SUPPORTING INFORMATION FOR PUBLICATION

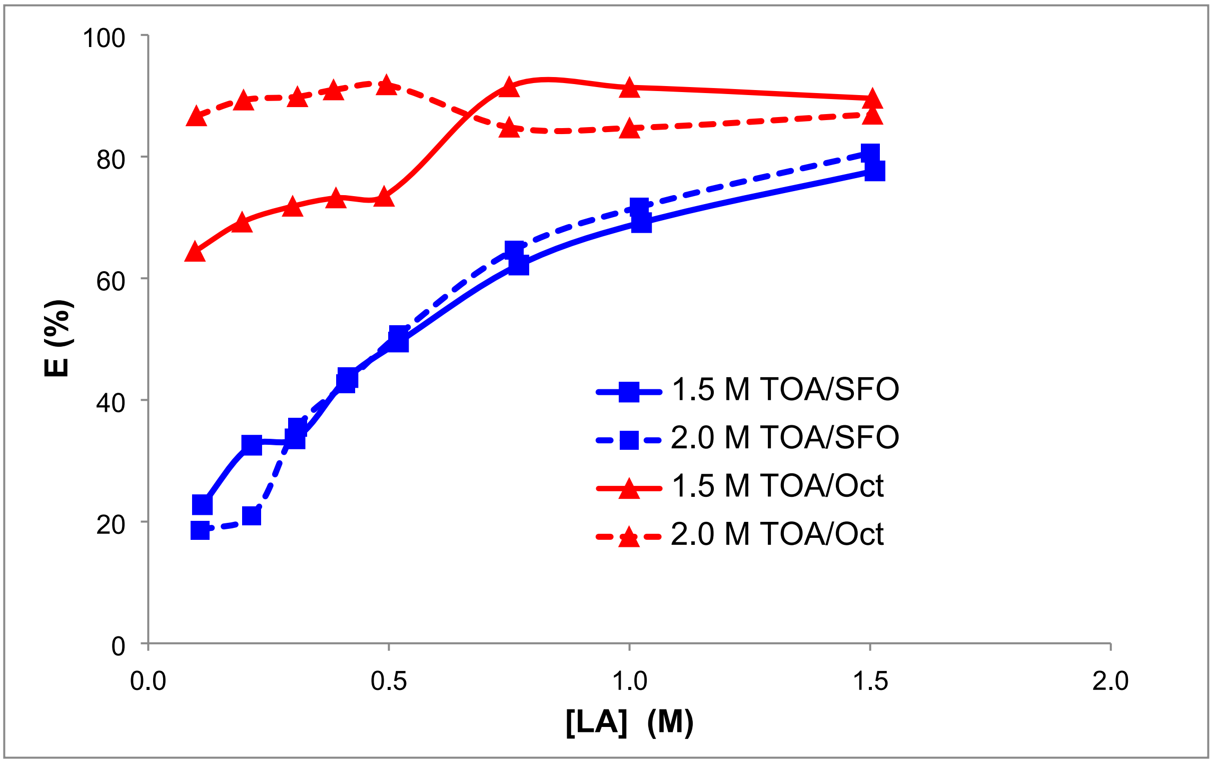

Figure S1. The effect of initial acid concentration on the complex extraction of LA at extreme TOA molarities (1.5 M and 2.0 M) in SFO and 1-octanol.
